# Protocol for a prospective observational study of conventional treatment and traditional Korean medicine combination treatment for children with cerebral palsy

**DOI:** 10.1186/s12906-016-1161-6

**Published:** 2016-06-08

**Authors:** Jeong-Eun Yoo, Young-Ju Yun, Yong-Beom Shin, Nam-kwen Kim, Soo-Yeon Kim, Myung-Jun Shin, Sun-ae Yu

**Affiliations:** Dunsan Korean Medicine Hospital of Daejeon University, Daejeon, South Korea; Department of Integrative Medicine, School of Korean Medicine, Pusan National University, 20 Geumo-ro, Yangsan, 626-789 Gyeongnam South Korea; Department of Rehabilitation Medicine, Pusan National University School of Medicine and Biomedical Research Institute, Pusan National University Hospital, Pusan, South Korea; Center for Comparative Effectiveness Research & Economic Evaluation in Korean Medicine, Pusan National University, Yangsan, South Korea; Department of Rehabilitation Medicine, Pusan National University Yangsan Hospital, Pusan National University School of Medicine, Yangsan, South Korea; Ulsan Korean Medicine Hospital of Dongeui University, Ulsan, South Korea

**Keywords:** Cerebral palsy, Rehabilitation, Gross Motor Function Measure (GMFM), Paediatric Evaluation of Disability Inventory (PEDI), Traditional Korean medicine (TKM)

## Abstract

**Background:**

Cerebral palsy leads to many complications as well as delayed motor development, and early intensive rehabilitation in infancy, which is based on the theory of brain plasticity, is emphasized. In addition to conventional treatment, including physical, occupational, or speech-language therapies, children also have a demand for traditional Korean medicine interventions such as acupuncture or herbal medicine; however, a lack of evidence has made traditional Korean medicine difficult to implement in practice. We planned a multicentre, prospective, observational study to assess the effectiveness, safety and cost-effectiveness of conventional treatment and traditional Korean medicine combination treatment for children with cerebral palsy.

**Methods/Design:**

Three hundred children with cerebral palsy aged 6 to 78 months will be recruited from six institutions. Data from each child are collected every month for a one-year period, during which time treatment might be changed or discontinued. A qualified investigator visits the sites to measure effectiveness variables, including Gross Motor Function Measure and Paediatric Evaluation of Disability Inventory. Adverse events and cost-effectiveness variables are collected using surveys conducted at baseline, mid-study, and end of study, as well as monthly tracking surveys. In the analyses, participants will be classified into two groups: group A children will be the conventional treatment group with physical, occupational, speech-language or other conventional rehabilitation therapies, whereas group B children will be the combination treatment group with traditional Korean medicine interventions, that is, herbal medicine, chuna, moxibustion and acupuncture, in addition to conventional treatment.

**Discussion:**

Only a few clinical case reports have evaluated the effectiveness and safety of traditional Korean medicine; therefore, more data are required to provide optimal information to children with cerebral palsy and their guardians. We hypothesized that traditional Korean medicine combination treatment for children with cerebral palsy would have benefits compared with conventional therapy alone. The findings of this study might provide informative data for conducting economic evaluations and developing clinical research on combination treatment for cerebral palsy in South Korea.

**Trial registration:**

NCT02223741

## Background

Cerebral palsy (CP) describes a group of disorders of the development of movement and posture that cause activity limitations; these disorders are attributed to non-progressive disturbances that occur in the developing foetal or infant brain [1]. Although the main feature of CP is a movement disorder, difficulties with cognition, learning, communication and behaviour often accompany CP. To manage these complicated situations, integrative therapies for rehabilitation have been developed and applied in practice [2]. From a systematic review of 49 studies, the pooled overall prevalence of CP was 2.11 per 1000 live births [3]. The prevalence of CP is 2.6 per 1000 children, and the attributable lifetime cost of CP is approximately 1.8 times higher than the basic lifetime medical cost of the general population in South Korea [4].

Intensive rehabilitation for young children is predicted to give potential benefits in terms of brain plasticity. Neuronal plasticity is enhanced in the developing brain, and it is usually adaptive and beneficial for neurological disorders such as CP. Clinical examples of adaptive neuronal plasticity include reorganization of cortical maps of the fingers in response to practice playing a stringed instrument and constraint-induced movement therapy to improve hemiparesis caused by CP [5].

In general, interventions include not only surgery or botulinum toxin for the inhibition of spasticity but also physical therapy (PT), occupational therapy (OT), speech therapy (ST), hydrotherapy, and other therapies [6]. Although there are public demands for complementary and alternative medicine (CAM) for children with CP, the lack of evidence makes alternative medicine difficult to implement in practice [7].

In a systematic review of traditional Chinese medicine (TCM) for children with CP, the authors concluded that a combination of either acupuncture plus chuna or acupuncture plus herbal medicine with conventional therapy showed significant beneficial effects on comprehensive function in terms of both physical and cognitive aspects, independence, and verbal function compared with conventional therapy alone [8]. Additionally, a rehabilitation approach including acupuncture that was intensely administered to young children with spastic CP resulted in significant functional improvement [9].

In Korea, children usually receive conventional treatment, including physical, occupational, or speech-language therapies, and they also have a demand for traditional Korean medicine (TKM) interventions such as acupuncture or herbal medicine. We now aim to investigate the effectiveness, safety, and cost-effectiveness of both conventional and TKM combination treatment for children with CP.

## Methods/Design

### Study design

**Fig. 1:**
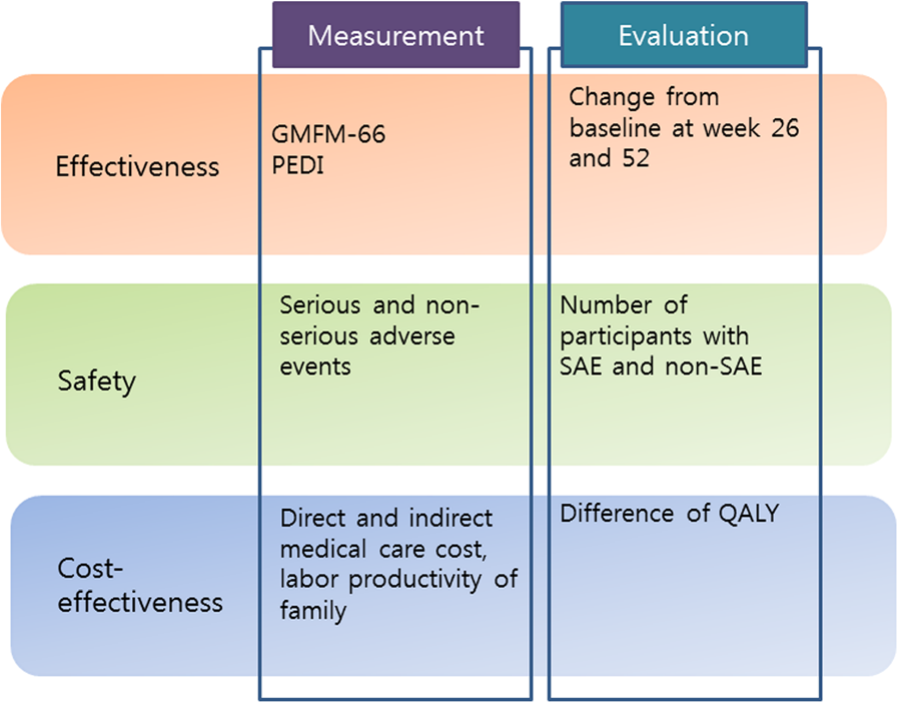
Observational study concept diagram. GMFM-66, Gross Motor Function Measure-66; QALYs, quality-adjusted life years

The study team is conducting a one-year, multi-site, prospective observational study of rehabilitation for children with CP. We are recruiting the children at two university-affiliated hospitals, one TKM hospital and three TKM clinics. The children with CP receive treatments from doctors and therapists in each clinic or centre, and one independent investigator assesses functional changes in participants and collects survey documents from their guardians or parents. We will analyse changes in the children’s functional development and health condition, the number of adverse events, and monetary resources spent for rehabilitation during this period. This design is a comparative observational study of a conventional treatment group and a TKM combination treatment group (Fig. 1).

### Participant eligibility

The study subjects are children with CP aged 6 to 78 months. Because the Paediatric Evaluation of Disability Inventory (PEDI) assessment module is suitable for children under the age of seven, we defined the upper age criterion considering the one-year follow-up period. Children who have had orthopaedic surgery due to CP; children who have congenital muscular disease, hereditary disease, or progressive central nervous system diseases; and children who suffer from severe diseases (i.e., cancer, severe heart disease, or severe infectious disease) are excluded from this study.

### Consent and registration

The subjects are being recruited from hospitals and local clinics specializing in CP. The children’s guardians provide consent to the enrolment because children under the age of seven cannot provide voluntary agreement. After the guardians are provided with study information leaflets, they have the opportunity to ask questions concerning the study. They are assured that participation or non-participation in the study will not affect the clinical care their children receive. Once informed consent is obtained, eligibility criteria are evaluated, and the central research group assigns each eligible participant an identification number. Registration of this study will be closed when the planned number of participants has been reached.

### Treatment

The participants undergo rehabilitation according to their doctors’ clinical care, but they do not receive any intentional treatments. During the period, they can freely start, cease or change any conventional treatments or TKM treatments. The conventional treatments can include PT, OT, ST and other conventional therapy, and the TKM treatments include herbal medicine, chuna, moxibustion and any type of acupuncture, e.g., body acupuncture, scalp acupuncture and pharmacopuncture. The most frequent set of TKM treatments implemented for CP children in South Korea is acupuncture and/or herbal medicine.

### Data collection

Data are obtained by participant survey and investigator measurement at baseline and at specified follow-up times (Table 1).

**Table 1:** Schedule of observations

	Screening	Registration	Observation period											
Study month (month)		baseline	1	2	3	4	5	6	7	8	9	10	11	12
Visit number	−1	1						2						3
Visit windows (week)							26 ± 1						52 ± 1	
Informed consent	●													
Demographics	●													
Medical history	●													
Inclusion/exclusion criteria	●													
Height		●						●						●
Weight		●						●						●
Concomitant disorders		●						●						●
Health problems		●						●						●
Survey														
Medication		●	●	●	●	●	●	●	●	●	●	●	●	●
Conventional treatment		●	●	●	●	●	●	●	●	●	●	●	●	●
TKM treatment		●	●	●	●	●	●	●	●	●	●	●	●	●
Health supplements		●	●	●	●	●	●	●	●	●	●	●	●	●
Medical care cost		●	●	●	●	●	●	●	●	●	●	●	●	●
Quality of life		●	●	●	●	●	●	●	●	●	●	●	●	●
Adverse events		●		●	●	●	●	●	●	●	●	●	●	●
Assessment														
GMFCS		●						●						●
GMFM-66		●						●						●
PEDI		●						●						●

●: assessment and observation will be performed

*TKM* traditional Korean medicine, *GMFCS* Gross Motor Function Classification System, *GMFM-66* Gross Motor Function Measure-66, *PEDI* Paediatric Evaluation of Disability Inventory

#### Participant survey

Parents or guardians respond to survey questionnaires. Data are collected using surveys conducted at baseline, mid-study, and end of study, and monthly tracking surveys will also be used. A baseline interview survey consists of basic characteristics, cause of CP, concomitant disorders, health problems, surgical history, medication, rehabilitation therapies, TKM treatment, health supplements, and medical care cost. An interview survey conducted on the 26th and 52nd weeks will include questions on concomitant disorders and health problems. The monthly tracking survey obtains information on health problems, changes in rehabilitation and treatments, medical care cost, and quality of life (QOL) with the Peds-QL CP module (ver 3.0) [10]. These guardian-reporting documents are collected and managed by the Clinical Research Center of Pusan Korean Medicine Hospital each month for a year. After the parents or guardians complete the forms, the investigators ensure that there are no blank spaces. In the case of insufficient data, we will check the accuracy of the information by reviewing medical records.

We will translate the PedsQL CP module (ver 3.0) into a Korean version and perform concurrent validation and reliability studies.

#### Investigator assessment

A qualified investigator visits the sites to measure each participant’s height and weight and classify each child according to the Gross Motor Function Classification System (GMFCS). The investigator is not a treatment provider nor an employee of any hospital or clinic, and this constitutes unbiased evaluation.

Gross motor function is assessed using the Gross Motor Function Measure-66 (GMFM-66) and the PEDI. All measurements and assessments will be performed at baseline and at the 26^th^ and 52^nd^ weeks. In this study, we will use the Korean versions of the GMFCS [11], GMFM-66 [12], and PEDI [13], which have undergone validity and reliability studies [14].

### Primary outcome measurement

The first primary outcome is the changes from baseline of the GMFM-66 scores at weeks 26 and 52. The GMFM-66 includes 66 items identified through Rasch analysis, which collectively best describe gross motor function in children with CP of varying abilities [15, 16]. The second primary outcome is the changes from baseline of the PEDI scores at weeks 26 and 52. The PEDI uses 217 questions to measure abilities in the three functional domains of self-care, mobility and social function [17].

### Secondary outcome measurement

The secondary outcomes involve safety and cost-effectiveness (utility) analysis components. One secondary outcome will be the number of participants with serious adverse events (SAEs) and non-SAEs during the study period. Cost data include direct medical and non-medical costs, indirect costs and productivity losses. Utility scores (EQ-5D) will be estimated from the mapping equation of PedsQL developed by Khan et al. [18], and then will be used for calculating quality-adjusted life years (QALYs) based on the area under the curve method. Cost-utility analysis will be conducted from a societal perspective, which includes direct medical costs, direct non-medical costs, and indirect non-medical costs measured as productivity loss of parents and other caregivers.

### Other measurements

Additional measurements of changes from baseline are evaluated, including height and weight at weeks 26 and 52, and changes in severity of concomitant disorders and health problems at weeks 26 and 52.

### Rationale for sample size

The sample size was determined on the basis of the primary outcome measure, GMFM-66. Using a 2-sided test, a sample size of approximately 37 children per treatment would assure 80 % power to detect a difference of at least 1.6 points (α = 0.05) assuming a standard deviation of 2.2 and dropout rate of 0.2. This mean change of 1.6 points in GMFM-66 scores was considered a clinically meaningful change in previous studies [17, 19]. We also planned the sub-group analyses considering disease severity measured by GMFCS (2 sub-groups, I-III and IV-V) and age (2 sub-groups, <3 and ≥3) heterogeneity. Therefore, a total of 300 participants will be required.

### Statistical and analytical plans

**Fig. 2:**
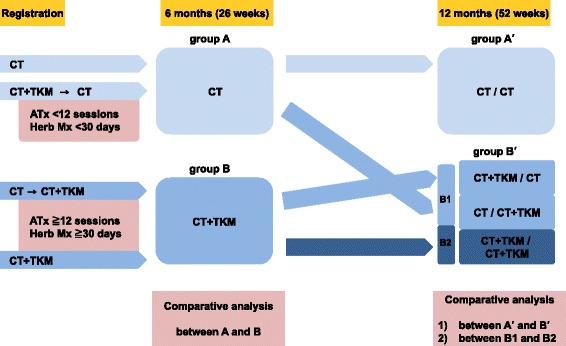
Process and time line of the observational study. CT, conventional treatment; TKM, traditional Korean medicine; ATx, acupuncture; Mx, medication

The study will present both descriptive and comparative results of conventional treatment and TKM combination treatment. In this study, we define operationally the conventional treatment (CT) group as children who have received only PT, OT, ST, and other conventional therapies, whereas the TKM combination treatment (CT + TKM) group is defined as children who have received at least twelve sessions of any type of acupuncture or 30 days of herbal medication within a 6-month follow-up period in addition to conventional therapies. At the time of analysis, participants will be classified in the conventional or TKM combination treatment group based on the six-month or one-year follow-up information (Fig. 2).

For the analysis of effectiveness, multiple variables, including age, severity of illness (indicated by the GMFCS), and treatments, will be converted to propensity scores by logistic regression, and the two groups will be compared by matching or stratification. We expect that using propensity scores will enhance the control variables of heterogeneous conditions. The last observation carried forward method will be used to handle missing data. The change from baseline of the GMFM-66 and PEDI scores at weeks 26 and 52 will be representative outcomes to evaluate effectiveness. These outcomes will be analysed by a two-sample *t*-test for the cross-sectional dataset and by a linear mixed model for the longitudinal dataset [20].

For sub-group analysis, we will analyse the correlation between rehabilitation intensity and functional changes in each group. We will also examine the different outcomes between acupuncture and acupuncture plus herbal medication cases in the TKM combination treatment group.

The number of participants with SAEs and non-SAEs will be reported via analysis of frequency, and the detailed progress of SAEs and related health problems will be disclosed as safety issues. In the case of long-term medication, we will carefully investigate adverse drug reactions using the drug-induced liver injury causality assessment scale [21].

The effectiveness of TKM reported by guardians will be used for further patient-centred outcome research. In the case of participants ceasing TKM treatments, we will investigate the reasons to enhance the feasibility of developing an RCT protocol.

Medical costs will be analysed for distribution characteristics, and we will perform a descriptive analysis of quantitative data. Each variable will be verified by statistical sensitivity and value analysis. Constant tariff values of the initial QOL measurements will be used to estimate QALYs between each follow-up interval, and a straight line of final QOL measurements will be used for extrapolation. An incremental cost-effectiveness ratio table and a cost-effectiveness plane will provide mean values of parameters, and a probabilistic sensitivity analysis will be conducted using distributions of estimated values of major variables. The analyses will be based on a societal perspective, and the discount rate will be 5 % based on the reference case recommendation of the Panel on Cost-Effectiveness in Health and Medicine and the Guidelines of Economic Evaluation of Medical Supplies in South Korea.

### Ethics and dissemination

The protocol of this study declares that participants will be protected against invasion of privacy, and the source data will be securely filed in the Clinical Research Centre for Korean Medicine. This study has been approved by the Institutional Review Board of Pusan National University Hospital (H1404-022-017). This study will be conducted with respect for the individual participants according to the Declaration of Helsinki, the Ethical Guidelines for Korea Good Clinical Practice, and relevant laws and regulations. The results of the present study will be published in a peer-reviewed journal and reported at relevant conferences.

## Discussion

A previous study reported that there is an increasing demand for CAM, especially traditional Korean medicine (TKM), among Korean children with CP [22]. TKM, which is derived from TCM, is very different from TCM in practice and is widely used in Korea. Only a few clinical case reports have evaluated the effectiveness and safety of TKM; therefore, more data are required to provide optimal information to children with CP and their guardians.

In general in South Korea, children with cerebral palsy concurrently receive their treatments in many rehabilitation clinics and rehabilitation centres in hospitals. Although they often change medical institutions for rehabilitation therapies, conventional therapies usually last approximately 30 min per session. Therefore, if we collect information regarding the types of interventions, the number of medical institutions, and the number of sessions, we can calculate the amount or intensity of intervention to a certain degree for statistical analysis.

The aim of this study is to analyse the effectiveness, safety, and cost-effectiveness of both conventional and TKM combination treatment for children with CP. This study is designed to evaluate the effectiveness of many types of interventions, including TKM, on cerebral palsy and to compare effectiveness between groups with or without TKM. From this initiative, we will attempt to identify the gross effectiveness of interventions, and then we will try to measure the amounts of interventions that can increase outcomes by digitizing intensity, duration, and frequency. We hypothesize that TKM combination treatment for children with CP will have benefits compared with conventional therapy alone, and as such, TKM combination treatment might be promoted in the development of an advanced guideline for integrative rehabilitation. Moreover, the findings of this study might provide informative data for conducting economic evaluations and developing a future randomized controlled trial (RCT) of combination treatment for CP in South Korea.

## Abbreviations

ATx, acupuncture; CAM, complementary and alternative medicine; CP, cerebral palsy; CT, conventional treatment; GMFCS, Gross Motor Function Classification System; GMFM-66, Gross Motor Function Measure-66; Mx, medication; OT, occupational therapy; PEDI, Paediatric Evaluation of Disability Inventory; PT, physical therapy; QALYs, quality-adjusted life years; QOL, quality of life; RCT, randomized controlled trial; SAEs, serious adverse events; ST, speech therapy; TCM, traditional Chinese medicine; TKM, traditional Korean medicine;
